# Supplementary material for: The Systems Biology Research Tool: evolvable open-source software
Source: BMC Syst Biol. 2008 Jun 29;2:55. doi: 10.1186/1752-0509-2-55 (PMC2446383; doi:10.1186/1752-0509-2-55)
Supplement: Additional file 1 — SBRT Archive. An archive of the current version of the Systems Biology Research Tool. [file 1752-0509-2-55-S1.zip › sbrt-1.4.0/doc/developers_guide/api/sbrt/kernel/math/geom/class-use/ConvexPolytope.html]

Uses of Interface sbrt.kernel.math.geom.ConvexPolytope


|  |  |  |
| --- | --- | --- |
| **Overview** \| **Package** \| **Class** \| **Use** \| **Tree** \| **Deprecated** \| **Index** \| **Help** | |  |
| PREV   NEXT | **FRAMES**    **NO FRAMES**     **All Classes** |  |


---


## **Uses of Interface sbrt.kernel.math.geom.ConvexPolytope**

| Packages that use ConvexPolytope | |
| --- | --- |
| **sbrt.kernel.math.geom** | Provides classes and interfaces for creating objects relevant to computational geometry. |
| **sbrt.kernel.math.geom.comp** | Provides classes and interfaces for performing geometry-related computations. |
| **sbrt.kernel.math.geom.proc** | Provides classes and interfaces for performing geometry-related processes. |

Uses of ConvexPolytope in sbrt.kernel.math.geom

| Classes in sbrt.kernel.math.geom that implement ConvexPolytope | |
| --- | --- |
| `class` | `HashConvexPolytope<V>`             This class is a concrete implemenation of `ConvexPolytope`. |
| `class` | `HashConvexPolytopeOld<V>`             This class is a concrete implemenation of `ConvexPolytope`. |

Uses of ConvexPolytope in sbrt.kernel.math.geom.comp

| Methods in sbrt.kernel.math.geom.comp that return ConvexPolytope | |
| --- | --- |
| `ConvexPolytope<V>` | `CdHarComputer.getPolytope()`             Returns the convex polytope for which random points are generated. |

Constructors in sbrt.kernel.math.geom.comp with parameters of type ConvexPolytope

`CdHarComputer(ConvexPolytope<V> polytope)`
  
          Constructs a new random point generator for the provided convex polytope.

Uses of ConvexPolytope in sbrt.kernel.math.geom.proc

Constructors in sbrt.kernel.math.geom.proc with parameters of type ConvexPolytope

`CdHarProcess(ConvexPolytope<V> polytope)`
  
          Constructs a new random point generator process for the provided polytope.

---


|  |  |  |
| --- | --- | --- |
| **Overview** \| **Package** \| **Class** \| **Use** \| **Tree** \| **Deprecated** \| **Index** \| **Help** | |  |
| PREV   NEXT | **FRAMES**    **NO FRAMES**     **All Classes** |  |


---
